# The impact of overweight on lipid phenotype in different forms of dyslipidemia: a retrospective cohort study

**DOI:** 10.1007/s40618-024-02368-5

**Published:** 2024-04-11

**Authors:** E. Formisano, E. Proietti, C. Borgarelli, S. G. Sukkar, M. Albertelli, M. Boschetti, L. Pisciotta

**Affiliations:** 1 Department of Internal Medicine, University of Genoa, Viale Benedetto XV 6, 16132 Genoa, Italy; 2 Dietetics and Clinical Nutrition Unit, IRCCS Policlinic Hospital San Martino, 16132 Genoa, Italy; 3 Endocrinology Unit, IRCCS Ospedale Policlinico San Martino, 16132 Genoa, Italy

**Keywords:** Obesity, Lipid phenotype, Familial dyslipidemias, Secondary dyslipidemias

## Abstract

**Purpose:**

Dyslipidemia plays a pivotal role in increasing cardiovascular risk. In clinical practice the misleading association between altered lipid profile and obesity is common, therefore genetically inherited dyslipidemias may not completely be addressed among patients with overweight. Thus, we aim to investigate the influence of overweight and obesity on the lipid phenotype in a cohort of patients with different forms of dyslipidemia.

**Methods:**

A retrospective analysis was conducted on patients with dyslipidemia from 2015 to 2022. Patients were stratified in familial hypercholesterolemia (FH), familial combined hyperlipidemia (FCHL), non-familial hyperlipidemia or polygenic hypercholesterolemia (PH). Clinical characteristics and lipid profile were evaluated.

**Results:**

Of the total of 798 patients, 361 were affected by non-familial hyperlipidemia (45.2%), while FCHL, FH and PH was described in 19.9%, 14.0% and 20.9% of patients, respectively. Overweight prevalence was higher in FCHL and non-familial hyperlipidemia patients than FH and PH patients. Subjects with overweight and obesity were independently associated with lower levels of high-density lipoprotein cholesterol (HDL-C) compared to patients with normal weight (52.4 and 46.0 vs 58.1, respectively; *p* < 0.0001); levels of triglycerides (TG) and non-HDL-C were higher in patients with overweight and obesity than patients with normal weight (257.3 and 290.9 vs 194.8, and 221.5 and 219.6 vs 210.1, *p* < 0.0001 and *p* = 0.01, respectively), while no differences were observed between patients with overweight and obesity.

**Conclusion:**

While dyslipidemias can be influenced by various factors, an important determinant may lie in genetics, frequently acting as an underlying cause of altered lipid profiles, even in cases of overweight conditions.

## Introduction

Dyslipidemia can be generally defined as a clinical condition characterized by a qualitative or quantitative alterations in plasma lipoproteins [1]. It is well established that dyslipidemia plays a crucial role in clinical practice due to its impact on increasing cardiovascular risk, making it one of the primary causes of atherosclerosis and its complications, including acute myocardial infarction, ischemic stroke, and peripheral arterial disease [2, 3].

Primitive dyslipidemias refer to genetically inherited conditions that can be categorized into monogenic and polygenic forms [4, 5]. Polygenic hypercholesterolemia (PH) is primarily attributed to impaired low density lipoprotein catabolism and is typically identified through clinical routine screening. The diagnosis of PH is based on clinical and anamnestic parameters, as there are no established dominant genetic alterations for this condition [6]. Familial Hypercholesterolemia (FH) appears to be the most prevalent condition among monogenic diseases, and FH patients present a higher cardiovascular risk, potentially leading to premature Coronary Artery Disease (CAD) [4, 7]. The clinical diagnosis of FH relies on the Dutch Lipid Clinic Criteria and it is characterized by high plasma levels of low-density lipoprotein cholesterol (LDL-C), usually associated with normal levels of plasma triglycerides (TG) and the presence of cholesterol deposits in peripheral cornea (corneal arcus) or in tendons and skin (xanthomas) [8, 9]. Familial Combined Hyperlipidemia (FCHL) is another common inherited dyslipidemia associated with premature CAD. Unlike FH, FCHL has a polygenic basis, involving more than 35 candidate genes, and it may present as mixed hyperlipidemia [10, 11]. In particular, FCHL presents increased and variable plasma levels of total cholesterol (TC) and TG, which are caused by hyperproduction of apolipoprotein B [12–14].

On the other hand, secondary dyslipidemias underline several ethiogenetic factors, including dietetical and lifestyle aspects, presence of comorbidities and drug therapy [15]. The difference from primary forms lies in the potential resolution of the lipid/lipoprotein abnormality once the underlying cause is corrected [16]. The most common conditions associated with dyslipidemias are hypothyroidism, primary biliary cholangitis, nephrotic syndrome, pregnancy, and medications such as antipsychotic, progestins, and diuretics [15, 17, 18]. Additionally, obesity, metabolic syndrome and an unbalanced diet are also implicated in the etiopathogenesis of secondary dyslipidemias [19].

It is well-known that dyslipidemia associated with obesity is characterized by an atherogenic lipid profile including higher levels of TG, with a reduction in high-density lipoprotein cholesterol (HDL-C) levels, along with an increased prevalence of small LDL particles [20, 21]. In clinical practice the misleading association between overweight and altered lipid profile is common, therefore the diagnosis of genetically inherited dyslipidemias may not completely be investigated among patients with overweight [22, 23]. Indeed, the influence of diet alone in improving the lipid profile seems to be unsatisfactory [24–26]. Therefore, it is essential to consider not only dietary cholesterol intake, but also its absorption through the biliary system, intestinal epithelial shedding, and endogenous synthesis [27]. Therefore, while dietary habits undoubtedly contribute to overall health, dyslipidemia appears to be influenced by a complex interplay of genetic and lifestyle factors [28].

Thus, the aim of our work was to investigate the influence of overweight and obesity on the lipid phenotype in a cohort of patients with different forms of dyslipidemia. Other aim was to assess the prevalence of primary and secondary dyslipidemias in our sample of patients.

## Methods

### Data collection and ranking methods

We conducted a retrospective cohort study including adult patients with dyslipidemia, with good thyroid function, followed by section of the Lipid Clinic of IRCCS Policlinic San Martino Hospital, University of Genoa, Italy, during the period from 2015 to 2022; patients in primary prevention attending the clinic, are requested to have a lipid profile test done without any ongoing lipid-lowering therapy for at least four weeks before their visit. As for patients in secondary prevention, lipid profile was documented under the condition of no ongoing therapy. Exclusion criteria were patients treated with concurrent lipid-lowering therapy and affected by diabetes. All included subjects underwent a medical evaluation: familiar, physiological, proximal and remote pathological anamnesis and smoking habits were taken into account; body mass index (BMI) and blood pressure were also assessed. We also collected waist circumference and fasting blood glucose when available. Blood tests performed within four weeks in a licensed laboratory without lipid-lowering treatment were evaluated and TC, HDL-C and TG levels were retrospectively recorded. The Friedewald formula was used to determine LDL-C, and non-HDL-C was calculated by subtracting HDL-C from TC. TG-Glucose (TyG) index was obtained according to the following equation: ln[TG × fasting blood glucose/2] [29].Gender, age, smoking habits, systolic and diastolic blood pression (SBP, DBP), weight, height, and BMI were registered. All patients were stratified according to their diagnosis in FH, FCHL, non-familial hyperlipidemia or PH. BMI was ranked in normal weight (BMI between 18,5 kg/m2 and 24,9 kg/m2), overweight (BMI between 25 kg/m2 and 29,9 kg/m2) and obesity (BMI ≥ 30 kg/m2). Informed written consent for using personal data for the present investigation was obtained from all the subjects. The study was conducted in accordance with the Declaration of Helsinki and was approved by the Ethics Committee of IRCCS Policlinic Hospital San Martino in Genoa, Italy (project numbers: 270/2020; 377/2023 DB id 13,324).

### Statistical analysis

Statistical analysis was performed using IBM SPSS Statistics, Version 25.0 (SPSS Inc., Chicago, IL, www.spss.com). A Kolmogorov–Smirnov analysis was performed to test the normality of variables. Ordinal and nominal variables, contingency tables were used for indicating frequency and percentage in the population. The results of continuous variables were expressed as median and interquartile range (IQR). For the comparison of continuous variables between different groups of patients, non-parametric tests of Kruskal–Wallis or Mann–Whitney were used when appropriate. Nominal variables were examined with the Pearson chi square (X2) test and with Spearman's rank correlation index for the correlation with continuous variables. Intergroup comparisons were adjusted for multiple comparisons with Bonferroni correction. Generalized multivariate model was used to adjust the parameters of the lipid profile, considering sex, age, BMI, and smoking habits as fixed factors and covariates.

## Results

A total of 798 patients affected by dyslipidemia were recruited in this retrospective study. Out of them, 41.7% were men (333/798), with a median age of 54 years (IQR 43, 63). The median BMI was 25.0 kg/m2 (IQR 22.7, 28.3), 446 patients (55.9%) were non-smokers and 344 subjects (43.3%) had hypertension.

Table 1 reports the patients’ characteristics stratified according to their diagnosis in FH, FCHL, Non-familial hyperlipidemia or PH. Most of patients were affected by non-familial hyperlipidemia (361/798, 45.2%), while FCHL, FH and PH was described in 158 (19.9%), 112 (14.0%) and 167 (20.9%) patients respectively.

**Table 1:** Patients characteristics stratified according to the diagnosis

	FCHL	FH	Non-familial	PH	p-value
Gender [F/M: n; %]	59 (37.3%) 99 (62.7%)	82 (73.2%) 30 (26.8%)	215 (59.6%) 146 (40.4%)	109 (65.3%) 58 (34.7%)	< 0.0001
Age *[years] *[median, IQR]	52 (40;70)	53 (40;64)	56 (46;66)	52 (43;61)	FCHL vs Non-familial: 0.003
BMI *[kg/m2]* [median, IQR]	26.1 (23.5;29.4)	24.1 (21.9;27.0)	25.4 (23.0;29.0)	24.0 (22.1;27.0)	< 0.0001
Normal weight [n; %]	59 (37.3%)	71 (63.4%)	162 (44.9%)	99 (59.3%)	FH vs Non-familial: 0.003 FH vs FCHL: < 0.0001 PH vs Non-familial: < 0.0001 PH vs FCHL: < 0.0001
Overweight [n; %]	65 (41.1%)	31 (27.7%)	125 (34.6%)	54 (32.3%)	
Obesity [n; %]	34 (21.5%)	10 (8.9%)	74 (20.5%)	14 (8.4%)	
Smokers [n; %]	39/158 (24.7%)	20/112 (17.9%)	77/361 (21.2%)	38/167 (22.8%)	0.300
Hypertension [n; %]	84/158 (53.2%)	37/112 (33.0%)	158/361 (43.8%)	65/167 (38.9%)	0.009
SBP [mm/Hg: median; IQR]	136 (113;151)	116 (110;114)	119 (113;150)	117 (111;150)	FH vs FCHL: 0.004

*BMI* body mass index. *DBP* diastolic blood pression, *FCHL* familial combined hyperlipidemia, *FH* familial hypercholesterolemia, *IQR* interquartile range, *PH* polygenic hypercholesterolemia, *SBP* systolic blood pression

Patients with FCHL were significantly more likely to be male (*p* < 0.0001), whereas patients with FH, PH, and non-familial hyperlipidemia were significantly more likely to be female (*p* < 0.0001). Patients with FCHL were significantly older than patients affected by non-familial hyperlipidemia (*p* = 0.003).

Patients with overweight were 275 (34.5%), while patients affected by obesity were 132 (16.5%). A diagnosis of inherited dyslipidemias was possible in 150 (54.5%) of patients with overweight, in 58 (43.9%) of patients with obesity and in 229 (58.6%) patients with normal weight with *p* = 0.014.

**Fig. 1:**
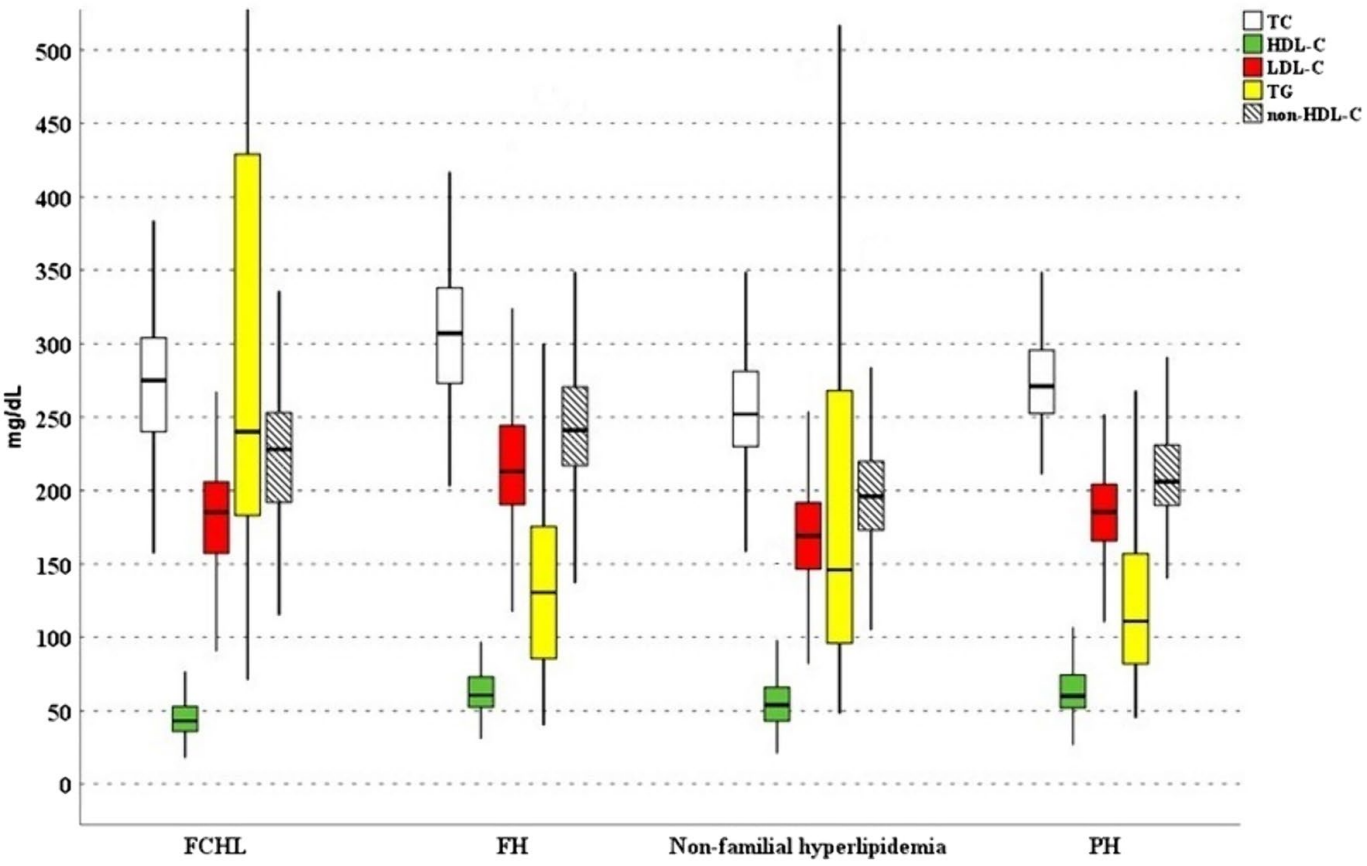
Diagnosis-related lipid profile distributions within our study population. This box plot representation compares the lipid profiles — Total Cholesterol (TC), High-Density Lipoprotein Cholesterol (HDL-C), Triglycerides (TG), and Non-High-Density Lipoprotein Cholesterol (Non-HDL-C) —across four distinct diagnostic categories: Familial Combined Hyperlipidemia (FCHL), Familial Hypercholesterolemia (FH), Non-familial hyperlipidemia, and Polygenic Hypercholesterolemia (PH). Each box plot illustrates the median (central line), interquartile range (box limits), and the full range excluding outliers (whiskers) for each lipid measure. The unit of measure was milligram on deciliter (mg/dL)

The prevalence of overweight and obesity were higher in patients with FCHL and non-familial hyperlipidemia than patients with FH and PH (Table 1). We did not find statistically significant differences for smoke habits between the four diagnoses of dyslipidemia, although patients with FH had significant lower levels of SBP compared to FCHL subjects (*p* = 0.004) and they were less likely to be diagnosed with arterial hypertension than patients with FCHL and non-familial hyperlipidemia. Figure 1 shows the diagnosis-related lipid profile of our population.

**Fig. 2:**
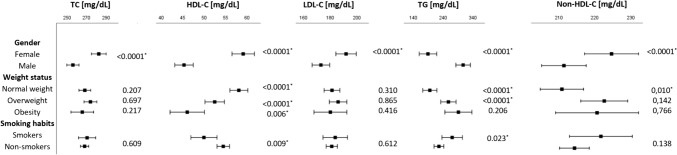
Cross-sectional multivariate analysis between lipid profile and gender, weight status and smoking habits. Each forest plot represents a different generalized multivariate model which was adjusted considering sex, age, BMI, and smoking habits as fixed factors and covariates. Each forest plot is completed with the p-value for included variables and * represents statistically significant differences. The value of lipid profile was reported as standardized mean for each factors and covariates. *HDL-C* high-density lipoprotein cholesterol, *LDL-C* low-density lipoprotein cholesterol, *TC* total cholesterol, *TG* triglycerides

A cross-sectional multivariate analysis was conducted to investigate the different lipid phenotype according to gender, weight status and smoking habits (Fig. 2).

Male gender was significantly associated with lower levels of TC (256.0, IC 95% 249.7–262.2 vs 282.6, IC 95% 275.0–290.1; *p* < 0.0001), HDL-C (45.3, IC 95% 43.1–47.4 vs 59.1, IC 95% 56.5–61.7; p < 0.0001), LDL-C (173.1, IC 95% 166.5- 179.7 vs 191.9 IC 95% 184.3–199.5; *p* < 0.0001), non-HDL-C (210.7, IC 95% 204.7–216.7 vs 223.5, IC 95% 216.2–230,74; *p* < 0.0001) and higher levels of TG than female patients (306.8, IC 95% 282.4–331.2 vs 188.6, IC 95% 159.0–218.1; p < 0.0001); active smokers were independently associated with lower levels of HDL-C (49.9, IC 95% 46.9–53.0 vs 54.4, IC 95% 53.0–55.9; *p* = 0.009) and higher levels of TG (269.9, IC 95% 235.5–305.4 vs 225.5, IC 95% 209.0–241.9; *p* = 0.023) than non-smokers.

Finally, subjects with overweight and obesity were independently associated with lower levels of HDL-C compared to patients with normal weight (52.4, IC 95% 50.2–54.7 and 46.0, IC 95% 42.0–50.0 vs 58.1, IC 95% 56.0–60.2, respectively; *p* < 0.0001); patients with overweight and obesity presented significant higher plasma levels of TG than subjects with normal weight (257.3, IC 95% 231.6–283.0 and 290.9, IC 95% 245.5–336.4 vs 194.8, IC 95% 171.2–218.5, respectively; p < 0.0001), while no significant differences were observed between subjects affected by overweight and obesity (257.3, IC 95% 231.6–283.0 vs 290.9, IC 95% 245.5–336.4, respectively; *p* = 0.206). Non-HDL-C was significantly higher in patients with overweight than patients with normal weight (221.5, IC 95% 215.2–227.8 vs 210.1, IC 95% 204.3–216.0; *p* = 0.010), while no significant differences were observed between patients with overweight and with obesity (221.5, IC 95% 215.2–227.8 vs 219.6, IC 95% 208.4–230.7, respectively *p* = 0.766).

Fasting blood glucose was available in 431 patients and its level was significantly different among patients with overweight (92.0 mg/dL, IQR 87.0–101.0), with obesity (95.0 mg/dL, IQR 90.0–103.0) and with normal weight (89.0 mg/dL, IQR 82–94) with *p* < 0.0001. Considering the TyG index, we observed an analogous behavior, as its value was higher in patients with overweight (9.06, IQR 8.58–9.57) and obesity (9.18, IQR 8.67–9.68) that subjects with normal weight (8.57, IQR 8.16–9.03) with *p* = 0.036.

In a subgroup of 213 patients, we also collected waist circumference as it was available in their medical records. This measure was directly associated with SBP (*r* = 0.153, *p* = 0.025), while no statistically significant associations emerged in lipid profile. We found a non-statistically significant association with higher levels of waist circumference in patients with non-familial dyslipidemias (105.0, IQR 98.0–109.0, *p* = 0.071) as compared to FH (100.0, IQR 96.0–107.0), FCHL (100.5, IQR 95.0–106.0) and PH (99.0, IQR 97–106).

## Discussion

Our study investigated the influence of overweight and obesity on the lipid phenotype in a cohort of patients with different forms of dyslipidemia. To date, most of the evidence is consistent with the finding that obesity could exacerbates dyslipidemia and contributes significantly to cardiovascular risk. Although dyslipidemias can be influenced by many factors, such as obesity, gender, and age, they are primarily genetic determinates. Then, lifestyle factors such as diet and physical activity play a role in managing dyslipidemias, it's crucial to recognize that genetic predisposition often underlies these conditions. In 2010, the Italian prevalences of overweight and obesity were 31.8% and 8.9%, respectively, but these data are progressively increasing [30, 31]. We included 34.5% and 16.5% of patients with overweight and obesity, respectively. This reflects a worldwide worrying growing trend, which requires targeted and incisive strategies.

Our findings highlight the different spectrum of hyperlipidemia among our patients and non-familial hyperlipidemia was the most prevalent, affecting nearly half of the patients (45.2%). This observation underscores the significant role that lifestyle and environmental factors can play in the development of dyslipidemias [32]. On the other hand, familial forms of hyperlipidemia, including FCHL, FH, and PH, collectively accounted for a substantial portion of cases. These results emphasize the genetic component inherent in some individuals' predisposition to hyperlipidemia. Familial forms of hyperlipidemia often carry a higher risk of cardiovascular complications, underlying the importance of early diagnosis and targeted management in affected individuals [33].

In our analysis, we observed an imbalance in the prevalence of individuals with overweight and higher BMI, favoring patients with FCHL and to a lesser extent, those with non-familial hyperlipidemia. Obesity-related dyslipidemia is traditionally linked to an unhealthy lifestyle and imbalanced diet. Despite being a common complication of obesity, it is often under-investigated diagnostically, frequently categorized as secondary rather than primary dyslipidemia [34]. However, differentiating between primary and secondary dyslipidemia can be challenging in some clinical scenarios. Indeed, a large number of patients with FCHL also exhibit metabolic syndrome, including factors like visceral obesity, multi-organ insulin resistance, non-alcoholic fatty liver disease, and hypertension, underlying this condition as an out-and- out multifactorial disorder [35].

In this context, we observed that patients with overweight and obesity were more likely to exhibit lower levels of HDL-C and non-HDL cholesterol. In the microenvironment of obesity, there is a complex interplay of factors that contribute to an altered lipid profile with a more atherogenic characteristics. This includes insulin resistance and the release of pro-inflammatory adipokines, which lead to fasting hypertriglyceridemia, decreased HDL-C levels, and the presence of smaller, denser LDL particles [36]. Obesity also makes the endothelium more permeable to atherogenic particles, promoting atherosclerosis [37]. Additionally, insulin resistance increases lipolysis and the production of circulating free fatty acids, while impaired elimination of lipoproteins exacerbates hypertriglyceridemia. This dyslipidemia, known as atherogenic dyslipidemia, is a significant risk factor for cardiovascular disease and often coexists with obesity in metabolic syndrome [38].

An intriguing finding from our analysis was the absence of a significant difference in TG levels between patients affected by overweight and obesity, whereas a significant difference was observed when compared to individuals with normal weight. Given that our population primarily consisted of patients with familial dyslipidemia, and among those about the 35% were affected by FH or PH, we hypothesize that these results may be influenced by genetic determinants of dyslipidemia. In fact, it is widely recognized that FH typically presents normal triglyceride levels, exhibiting a type IIa lipid profile and elevated triglyceride levels are an infrequent occurrence in this context [39]. Also, in cases of PH, individuals may exhibit serum triglyceride concentrations within the reference range, suggesting that the genetic factors influencing cholesterol levels might not always directly impact triglyceride concentrations [40]. The misdiagnosis of FCHL over FH or PH can occur due to overlapping clinical features and lipid profiles [41]. Genetic testing can indeed be a valuable tool in resolving such diagnostic challenges, but its clinical application may be limited by factors such as cost, accessibility, and the need for specialized expertise in genetic interpretation [42]. Additionally, genetic tests may not always identify all relevant genetic variants or account for environmental factors that can influence lipid disorders [43].

All in all, we feel that the presence of hypertriglyceridemia could mask a concomitant alteration of cholesterol and potentially leading to an underestimation of the real cardiovascular risk in patients. In fact, many diagnoses of secondary hyperlipidemia may overlap with primary hypercholesterolemic disorders characterized by unspecified high levels of triglycerides. Given this knowledge, we hypothesize that our findings might recall to the theory known as "obesity stigma” [44]. Consequently, this mindset could significantly impact the well-being of individuals with obesity, as they may encounter stigmatization, even from healthcare professionals. This may lead to a form of "therapeutic inertia", characterized by hesitation to request specific clinical investigations due to an overemphasis on signs and symptoms attributable to obesity [45]. On the other hand, we observed that the TyG index values were significantly higher in patients with overweight and obesity compared to those with normal weight. The differences in these metabolic markers suggest a potential increased risk of insulin resistance among patients affected by overweight and obesity [46].

The main limitation of the present study is the inherent bias of cross-sectional studies. Secondly, all participants involved were from a single center, which may limit the generalizability of our findings to other populations. Finally, unmeasured confounding might exist due to the partial unavailability of anthropometrical data, such as waist circumference and body composition, as well as the lack of complete information regarding physical activity and fasting blood glucose, which could potentially act as mediators in the relationship between different phenotypes of dyslipidemia and overweight. In this field, the absence of complete data on waist-to-hip ratio and the TyG index represents a limitation of our analysis and arises from the nature of our data collection. Indeed, it is well-known that the importance of these parameters in relation to dyslipidemias and their potential association with cardiovascular injury is pivotal [47, 48]. Additionally, we excluded patients with diabetes precluding the analysis of its potential influence on lipid profiles. Finally, in our study the enrollment of patients from a third-level specialist center at a University Hospital may have induced a selection bias.

Despite these limitations, the relatively large sample size analyzed and the use of appropriate analysis to balance confounding factors, enhance the strength of our findings.

## Conclusion

In summary, our analysis confirms that several factors, such as overweight, gender, and age, can contribute to the development of dyslipidemias, but the clinical diagnosis of inherited dyslipidemias may play a role in patients further characterization. On the other hand, our findings suggest that while overweight and obesity are prevalent and influential factors, they do not fully account for the dyslipidemia patterns observed, particularly in genetically predisposed populations. This highlights the importance of individualized medical approaches for diagnosis and treatment of dyslipidemias, which should consider both genetic, demographical, and anthropometrical factors to successfully manage cardiovascular risk associated with dyslipidemias.

## Data Availability

Data for the reported results are available upon a reasonable request, in accordance with ethical principles.

## Declarations

### Research involving human

All procedures performed in studies involving human participants were in accordance with the ethical standards of the institutional and/or national research committee and with the 1964 Helsinki declaration and its later amendments or comparable ethical standards.
